# Estimation of poverty bounds for Pakistan using synthetic panel data

**DOI:** 10.1371/journal.pone.0276673

**Published:** 2023-03-23

**Authors:** Nadia Shabnam, Waqar Ameer, Neelam Aurangzeb, Muhammad Azeem Ashraf, Syed Hasanat Shah

**Affiliations:** 1 Department of Health Profession Education, National University of Medical Sciences, Islamabad, Pakistan; 2 Department of Economics, Shandong Business and Technology University, Shandong, China; 3 Department of Mathematics and Statistics, International Islamic University, Islamabad, Pakistan; 4 Research Institute of Education Science, Hunan University, Changsha, China; 5 School of Economic, Jilin University, Jilin, People’s Republic of China; University of Pisa, ITALY

## Abstract

Poverty is a big threat to prosperity in developing countries like Pakistan. Alleviating poverty needs concerted efforts including how to measure and analyze poverty. Therefore, this paper employs synthetic panel technique and uses repeated cross-sections household survey dataset (Household Integrated and Economic Survey (HIES)) of Pakistan for 2010–11 and 2015–16, to derive poverty bounds for Pakistan. The findings of the paper suggest that 17% of population still remains in poverty in 2015–16 as they were in 2010–11. They don’t move in or out of poverty. In the same periods 19% population affected by poverty. The 2.5% poor’s of 2010–11 moves out of poverty in 2015–16. This constitutes the first attempt to provide an insight into poverty dynamics in Pakistan using the available survey data.

## 1. Introduction

Poverty is a global phenomenon. Poverty exists where people are unable to meet their basic needs or they do not get essentials for survival. World Bank define poverty in quantitative terms by asserting that a person is considered to be poor if they earn less than 1.90 US dollars per day [1]. The report on Global Multidimensional Poverty Index (MPI) by World Bank [2] estimated that 1.3 billion people around the world are still living in extreme poverty despite marked decline in global poverty. This report also showed that global extreme poverty fell from 36 percent (1.85 billion) in 1990 to 10 percent (0.736 billion) in 2015. It seems that more than a billion peoples moved out of extreme poverty during the course of 25 years from 1990 to 2015. Pakistan also reported a declining trend in poverty in the same period of time where poverty on national level declined from 59.4 percent in 2005–06 to 24.3 percent in 2015–16 (Table 1).

**Table 1:** Poverty trends in Pakistan, 2005–06 to 2015–16.

Year	National	Urban	Rural
2005–06	50.4	36.6	57.4
2007–08	44.1	32.7	49.7
2010–11	36.8	26.2	42.1
2011–12	36.3	22.8	43.1
2013–14	29.5	18.2	35.6
2015–16	24.3	12.5	30.7

Source: Pakistan Economic Survey (2015–16) [3]

Poverty is not only a major cause of social tension and development but at the same time it also a big threat to human dignity and civilization. Therefore, concerted efforts are required to tackle the menace of poverty. Pakistan is an under developing country where poverty remained high [4]. Luckily poverty is on decline in Pakistan, however, still there is a lot to be done to tackle the issue of poverty. The 2018 MPI report [2] consider that the first step to tackle poverty is to acquire accurate data and employ appropriate methods.

Some studies [5] rigorously analyzed poverty from different aspects but they ignored the role of time in their analysis. The static measure of poverty may assign equal weight to people who have experienced poverty for the first time and those who are in perpetual poverty. Static analysis of poverty make it difficult to understand whether the observed poor persons of current year is different from the observed poor in last year?” In other words static analysis fails to offer a complete picture of poverty and thus impede policy efforts. Globally, policies to alleviate extreme poverty differ across space. Effectiveness of policies also depend on the nature of poverty i.e. chronic or transitory. Moreover, we must focus and understand the dynamics of poverty in order to help an individual to escape poverty. One way to do it is to collect quality data, especially panel data. The collection of panel data can be a challenging task for a country and it may be costly but use of panel data is very effective to measure the intensity and nature of poverty. For a variety of reasons, the pseudo panel data are more common in most of the developing countries compared to panel data.

To overcome the non-availability of data, number of studies in the literature [6, 7] started to develop pseudo panel data out of multiple rounds of cross sectional data as well as cohort level mobility to explore the dynamics of income and consumption over time. The use ability of cohort-means impedes the examination of income mobility at a level more disaggregated than that of the cohort [8]. They have [8] also construct the poverty map and showed how repeated cross-sectional data of household survey can provide inference about transition of poverty. They estimated upper and lower bounds of poverty by using parametric and non-parametric approach and then compared results with original data measures. Two survey data VHLSS (2006 & 2008) and IFLS (1997 & 2000) was used. These synthetic panels were used to predict lower and upper bound estimates of household transitions into and out of poverty in Vietnam and Indonesia. The aim of his study was to develop the method that able to estimate poverty mobility and immobility at same level that would be estimated in the genuine panel. The results show that parametric approach estimates provide better analysis of household consumption and cross-sectional way of analysis is more reliable and less costly than panel.

In this framework, we have tried to do the analysis of poverty in Pakistan proposed by [7] The main objectives of this study are: 1) to derive and estimate the poverty bounds through Parametric and Non-Parametric Regression methods. 2) to explore the prospects of some insights into mobility and immobility and poverty duration and to test the performance of different interval length.

We employed non-parametric and parametric bounding methods in our investigation to compare the performance of the methods in real-world situations. The data and the model’s underlying error distribution are not subject to any restriction when using a non-parametric technique. The width of the bound in non-parametric approach depends on the extent to which the time invariant regressors explain cross sectional consumption. Moreover, even when the precise autocorrelation is unknown, information from other data sources may often be available that points to a much smaller (and well-known) range than the range between zero and one as the genuine autocorrelation. We offer a parametric bounding method that can be applied in these situations; it involves additional assumptions but allows for a narrowing of the bounds in comparison to the non-parametric method.

The remainder of the paper is structured as follows: Section 2 provides a brief literature review which is then followed by the section 3 having theoretical framework for obtaining bounds on movements into and out of poverty. Sections 4 and 5 describes the parametric and nonparametric estimation methods and data description respectively. Section 6 examines robustness to the choice of poverty line and provides an application to profiling of poverty dynamics. Section 7 concludes. Section 8 describes the limitations.

## 2. Literature

Poverty is a big challenge for the process of development and social integrity. For many decades, there was wide debate on measuring and analyzing poverty dynamics. The current literature has showed consents that poverty has multidimensional facts [9]. The income or calories base measurement of poverty considered only one-dimension and ignores the all other important aspects of poverty transition [4, 10, 11]. The literature encompasses poverty dynamics under two primary approaches. One is Spell approach [8, 12–15] and other is component base approach [16, 17] methods. The spell-based method focuses on the household movement up and down back across poverty on the base of income or consumption. It does not capture all poverty dynamics below the poverty lines. It also ignores the degree of transition and measurement of chronic poor. Furthermore, one of the study [18] also provides four more concept regarding on the base of spell-based approach. He categorized four forms of poverty over time such as: Always poor: observed permanently consumption expenditure below poverty line. Usually poor: non poor but at least one poverty period threshold observed. Churning poor: Living standard over times fluctuates, they move out of poverty as much as they could stay in. Occasionally poor: They experienced a poverty spell but within a short time stay out of poverty line.

The authors [6] developed the pseudo panel data, from multiple rounds of cross-sectional data. The synthetic panel was constructed based on age cohorts across many surveys allowable to provided details information on consumption and income. The study [7] pioneered the methodology based on the repeated cross-sectional data by using second order moment approach for United States. He used the estimates obtained from individual vulnerability to poverty at a point in time on the base of cross-sectional data information. Similarly some studies [19] used pseudo panel method to assess poverty trends and existence of linkages between poverty and gender in South Africa. They analyzed birth cohort on three levels by using Living Conditions Surveys (LCS) of 2008–9, Income and Expenditure Survey (IES) of 2010 and 2014. The first level compared the per capita expenditure mean between birth cohorts 2008 to 2014. On second level multiple regression conducted to separate age, cohort effects and time. In last section they analyze poverty for birth cohorts. Results demonstrate poverty affect birth cohorts of old ages more than it affects birth cohorts of the younger ages. Another study [20] discussed the use of the synthetic panels approach and applied on intra-year panel survey data 2014–15 for Mozambique. They analyzed that higher percentage of population remaining same in the given year either in or out of poverty and smaller percentage moving upward or downward across poverty line. Results from synthetic panels are close to the true values estimated using the panel data of 2014–15. They find high variability in consumption in Mozambique and reflect a big change in intra-year of poverty dynamics.

Despite a pressing issue for Pakistan, a few studies focused on poverty transition and dynamics in Pakistan. For example, [21] used panel data to examine poverty transition in Pakistan. He concluded that 25 percent of poorest peoples move out of poverty from 1998–99 to 2000–01. Similarly, [22] estimates poverty measures incidence along with depth and severity for rural and urban poverty. He used consistent methodology and computes national and regional poverty lines for Pakistan. He concluded that 38 percent population of Pakistan live below the poverty line. Another study from Pakistan [23] observed the poverty in the rural areas of Pakistan (Sindh & Punjab) by using logistic regression approach. They concluded that 11 percent of Pakistan population is experiencing chronical poverty from 2001 to 2004.

## 3. Theoretical bounds and synthetic panel approach

This section provides the brief overview of methods that used to construct the synthetic panel, as well as upper and lower bounds of poverty for Pakistan. The study considered two cross sectional surveys (2010–11 and 2015–16), consist household sample N1 and N2 respectively. These survey rounds are denoted by round 1 and round 2. We assumed that both survey samples are random samples of the underlying population.

The linear projection of round 1 consumption, *y*_*i*1_ onto *x*_*i*1_ is given by:

$$y_{i1}=\alpha_{1}^{'}x_{i1}+\mu_{i1}$$ (1)

Let *x*_*il*_ be a vector describes the i^th^ household characteristics in survey rounds 1. These characteristics are observed in both survey rounds for different household, includes time invariant characteristics of household head (e.g., education, residence, occupation status, birth, gender and deterministic characteristics such as age etc.) and household (e.g., language, birth place, education religion, region etc.). It also includes time varying characteristics of household such as unexpected household shocks.

Letting, *y*_*i*1_ and *μ*_*i*1_ denotes the per capita expenditure and error term in survey round 1.

The linear projection of round 2 consumption, *y*_*i*2_ onto *x*_*i*2_ is given by:

$$y_{i2}=\alpha_{2}^{'}x_{i2}+\mu_{i2}$$ (2)

Let *x*_*i*2_ also denote the set of household characteristics for survey round 2 that observed in both survey rounds.

Additionally, to measure inference about the movement in and out of poverty depended on household consumption expenditure in round 2 and estimated expenditure of same household in round 1. This mobility degree is estimated by:

$$p(y_{i1}<z_{1}\cap y_{i2}>z_{2})$$ (3)

Eq (3) represents the household degree of ‘movement out of poverty’ over two survey rounds. The difficulty facing with repeated cross-sections is that it does not provide the information of household consumption expenditure for the same households in both survey rounds [8]. However, it is possible to obtain the upper and lower bounds of poverty. To derive these bounds we can rewrite the probability in Eq (3) as:

$$P(\mu_{i1}<z_{1}-\alpha_{1}^{\prime }x_{i1}\cap \mu_{i2}>z_{2}-\alpha_{2}^{\prime }x_{i2})$$ (4)

This probability depends on the joint distribution of error terms μ_*i*1_ and μ_*i*2_. These error terms capturing the correlation of those parts of household consumption expenditure in two survey rounds which are not captured by *x*_*i*1_ and *x*_*i*2_. The notations *z*_1_ and *z*_2_ defines the poverty line in round 1 and 2 respectively. We further need two data assumptions to operationalize the proposed methodology [7].

### 3.1. Assumption 1

The underlying population is same in both survey periods. This assumption overcome the absence of panel data and ensures the used of time invariant household characteristics that are observed in both survey periods to measure the household predicted consumption. Assumption 1 fails to fulfil the sample requirement if underlying population is changed through death, migration or birth out of sample. Assumption 1 also fails due survey related technical issues such as change is sampling methodology from round 1 to round 2 [24].

### 3.2. Assumption 2

This assumption is about the existence of correlation between error terms (μ_*i*1_, μ_*i*2_). We assume that error terms are positive quadrant dependent (PDQ), which entails that correlation between error terms is non-negative. There are mainly three reasons on the basis of which this assumption considered to be appropriate for the household survey data. Firstly, as the household poverty status has a tendency to be strongly related over time, the joint probability that a household is poor in both survey rounds considered together is expected to be higher than the product of the probability that this household is poor in first and second round, respectively. The joint probability is considered to be chronic poverty rate and the latter product probability is the poverty rates computed from the repeated cross sections.

Secondly, economic shocks–such as finding or losing a job–have some perseverance and expenditures always reacts to these economic shocks. It means consumption errors will also reveal positive autocorrelation. Lastly, a negative correlation can be observed in income over the survey rounds for some particular households, which are implausibly apply to the whole population at the same time due to some unappealing factors. For instance, a household that lacks access to credit may cut the expenses in round one in order to pay for an important event in round 2. For such kind of households, we would observe a lower consumption than their x variables would predict in round one, and higher consumption than would be predicted for round two.

In a standard pseudo panel analysis or synthetic panel data analysis these two assumptions will be carried out by imposing condition on the household headed by people aged 25 to 55. Analysis of poverty transition of Households headed by those younger than 25 or older than 55 is more difficult, the reason being that in these age groups either households are beginning to form or starting to dissolve.

### 3.3. Derivation of poverty upper bound estimates

According to assumption 1 and 2, the upper bound estimates of poverty given by probability in Eq (4), when error terms (μ_*i*1_, μ_*i*2_) are independent to each other. It means Correlation between error terms (μ_*i*1_, μ_*i*2_) = 0. In this situation, the following equations shows the upper bounds estimates for poverty mobility:

$$P(y_{i1}^{2U}<z_{1}\cap y_{i2}>z_{2})=P(\mu_{i1}<z_{1}-\alpha_{1}^{\prime }x_{i2})P(\mu_{i2}>z_{2}-\alpha_{2}^{\prime }x_{i2})$$ (5)

Eq (5) used to estimates household movement out of poverty, $y^{2U}{}_{i1}$ here the superscript 2 defines household estimates round 1 consumption for household sampled in round 2 and U stands for the upper bound estimates of poverty mobility. For movements into poverty, the Eq (6) are as follows

$$P(y_{i1}^{2U}>z_{1}\cap y_{i2}<z_{2})=P(\mu_{i1}>z_{1}-\alpha_{1}^{\prime }x_{i2})P(\mu_{i2}<z_{2}-\alpha_{2}^{\prime }x_{i2})$$ (6)

Eq (6) used to estimates household movement into poverty, these two equations derive as:

#### • Proof 1

The probability of the household movement out of poverty in second round of period can be written as:

$$P(y_{i1}<z_{1}\cap y_{i2}>z_{2})=P(\mu_{i1}<z_{1}-\alpha_{1}^{\prime }x_{i1}\cap \mu_{i2}>z_{2}-\alpha_{2}^{\prime }x_{i2})$$

$$=P(\mu_{i1}<z_{1}-\alpha_{1}^{\prime }x_{i2}\cap \mu_{i2}>z_{2}-\alpha_{2}^{\prime }x_{i2})$$

$$=P(\mu_{i1}<z_{1}-\alpha_{1}^{\prime }x_{i2})P(\mu_{i2}>z_{2}-\alpha_{2}^{\prime }x_{i2}|\mu_{i1}<z_{1}-\alpha_{1}^{\prime }x_{i2})$$ (7)

Using of assumption 1 condition, second line follows by replacing *x*_*i*1_ with *x*_*i*2_ while the third line follows multiplication rule for conditional probabilities.

It follows that

$$P(\mu_{i2}>z_{2}-\alpha_{2}^{\prime }x_{i2}|\mu_{i1}<z_{1}-\alpha_{1}^{\prime }x_{i2})\leq P(\mu_{i2}>z_{2}-\alpha_{2}^{\prime }x_{i2})$$ (8)

Strictly speaking, we required $P(\mu_{i1}<z_{1}-\alpha_{1}^{\prime }x_{i2})$ > 0 to derive the third line, which is satisfied as long as the poverty rate is not zero for period 1. As (*μ*_*i*1_, *μ*_*i*2_) are positive quadrant dependent, mentioned in assumption 2. Subtract the both sides of Eq (8) from $P(\mu_{i2}>z_{2}-\alpha_{2}^{\prime }x_{i2})$, we have

$$P(\mu_{i1}<z_{1}-\alpha_{1}^{\prime }x_{i2}\;\mathrm{and}\;\mu_{i2}>z_{2}-\alpha_{2}^{\prime }x_{i2})\leq P(\mu_{i1}<z_{1}-\alpha_{1}^{\prime }x_{i2})P(\mu_{i2}>z_{2}-\alpha_{2}^{\prime }x_{i2})$$ (9)

Then divided both sides by $P(\mu_{i1}<z_{1}-\alpha_{1}^{\prime }x_{i2})$ and we have stated the results

$$P(y_{i1}<z_{1}\cap y_{i2}>z_{2})\leq P(\mu_{i1}<z_{1}-\alpha_{1}^{\prime }x_{i2})P(\mu_{i2}>z_{2}-\alpha_{2}^{\prime }x_{i2})$$ (10)

The upper bound estimates can be written as:

$$P(y_{i1}^{2U}<z_{1}\cap y_{i2}>z_{2})=P(\mu_{i1}<z_{1}-\alpha_{1}^{\prime }x_{i2})P(\mu_{i2}>z_{2}-\alpha_{2}^{\prime }x_{i2}).$$ (11)

Thus combining Eqs (10) and (11), it follows that

$$P(y_{i1}^{2U}<z_{1}\cap y_{i2}>z_{2})\geq P(y_{i1}<z_{1}\cap y_{i2}>z_{2})$$ (12)

Which establishes the upper bound estimates of mobility. Then subtracting each of the term in Eq (12) from *P*(*y*_*i*2_>*z*_2_), we would have

$$P(y_{i1}^{2U}\geq z_{1}\cap y_{i2}>z_{2})=P(y_{i1}\geq z_{1}\;and\;y_{i2}>z_{2})$$ (13)

### 3.4. Derivation of poverty lower bound estimates

The lower bounds estimates of poverty mobility follows the assumption of data when two error terms totally dependent to each other, that is corr (μ_*i*1_, μ_*i*2_) = 1.

$$P(y_{i1}^{2L}<z_{1}\cap y_{i2}>z_{2})=P(\gamma \mu_{i2}<z_{1}-\alpha_{1}^{\prime }x_{i2})-P(\mu_{i2}\leq z_{2}-\alpha_{2}^{\prime }x_{i2})$$ (14)

for movement out of poverty, and

$$P(y_{i1}^{2L}>z_{1}\cap y_{i2}<z_{2})=P(\mu_{i2}<z_{2}-\alpha_{2}^{\prime }x_{i2})-P(\gamma \mu_{i2}\leq z_{1}-\alpha_{1}^{\prime }x_{i2})$$ (15)

for movement into poverty; where $y_{i1}^{2L}$ the superscript 2 stands for household consumption estimated in round 1 for the households sampled in round 2, L stands for the lower bound estimates of poverty mobility.

#### • Proof 2


$$\begin{array}{l} P(y_{i1}<z_{1}\cap y_{i2}>z_{2})=P(\alpha_{1}^{\prime }x_{i1}+\mu_{i1}<z_{1}\cap \alpha_{2}^{\prime }x_{i2}+\mu_{i2}>z_{2})\\ \;=P(\mu_{i1}<z_{1}-\alpha_{1}^{\prime }x_{i1})+P(\mu_{i2}>z_{2}-\alpha_{2}^{\prime }x_{i2})\\ \;-P(\mu_{i1}<z_{1}-\alpha_{1}^{\prime }x_{i1}\cup \mu_{i2}>z_{2}-\alpha_{2}^{\prime }x_{i2})\\ \;=P(\mu_{i1}<z_{1}-\alpha_{1}^{\prime }x_{i1})+[1-P(\mu_{i2}\leq z_{2}-\alpha_{2}^{\prime }x_{i2})]\\ \;-P(\mu_{i1}<z_{1}-\alpha_{1}^{\prime }x_{i1}\cup \mu_{i2}>z_{2}-\alpha_{2}^{\prime }x_{i2})\\ \;=P(\mu_{i1}<z_{1}-\alpha_{1}^{\prime }x_{i1})-P(\mu_{i2}\leq z_{2}-\alpha_{2}^{\prime }x_{i2})\\ \;+[1-P(\mu_{i1}<z_{1}-\alpha_{1}^{\prime }x_{i1}\cup \mu_{i2}>z_{2}-\alpha_{2}^{\prime }x_{i2})]\\ \;=P(\mu_{i1}<z_{1}-\alpha_{1}^{\prime }x_{i2})-P(\mu_{i2}\leq z_{2}-\alpha_{2}^{\prime }x_{i2})\\ \;+[1-P(\mu_{i1}\leq z_{1}-\alpha_{1}^{\prime }x_{i2}\cup \mu_{i2}>z_{2}-\alpha_{2}^{\prime }x_{i2})] \end{array}$$ (16)

Where second and third lines indicates the properties of probability, last lines follows the assumption 1: by replacing *x*_*i*2_ with *x*_*i*2_

$$\begin{array}{l} P(y_{i1}^{2L}<z_{1}\cap y_{i2}>z_{2})=P(\gamma \mu_{i2}<z_{1}-\alpha_{1}^{\prime }x_{i2}\cap \mu_{i2}>z_{2}-\alpha_{2}^{\prime }x_{i2})\\ \;=P(\gamma \mu_{i2}<z_{1}-\alpha_{1}^{\prime }x_{i2})-P(\mu_{i2}\leq z_{2}-\alpha_{2}^{\prime }x_{i2})\\ \;=P(\mu_{i2}<z_{1}-\alpha_{1}^{\prime }x_{i2})-P(\mu_{i2}\leq z_{2}-\alpha_{2}^{\prime }x_{i2}) \end{array}$$ (17)

Where $\gamma =\frac{{\hat{\sigma }}_{\mu 1}}{{\hat{\sigma }}_{\mu 2}}$ Last lines shows the perfect correlation between *u*_*il*_ and *u*_*i*2_,

$$Since,\;cor(u_{i1}{,\;u}_{i2})=\frac{cor(u_{i1}{,\;u}_{i2})}{\sqrt{{\hat{\sigma }}_{u_{1}}^{2}{\hat{\sigma }}_{u_{2}}^{2}}}=1,\;\mathrm{Its}\;\mathrm{follows}\;cor(u_{i1}{,\;u}_{i2})=\sqrt{{\hat{\sigma }}_{u_{1}}^{2}{\hat{\sigma }}_{u_{2}}^{2}}$$

$$Hence,\;\gamma =\frac{cor(u_{i1}{,\;u}_{i2})}{{\hat{\sigma }}_{u_{2}}^{2}}=\frac{{\hat{\sigma }}_{u_{1}}^{2}}{{\hat{\sigma }}_{u_{2}}^{2}}$$

## 4. Parametric and non-parametric approach

The pervious section provides the procedure, theoretical frame work and theorems to obtain the lower and upper bound of poverty. The assumptions about joint distribution of error term are important for poverty mobility measures. These assumptions can be violate on different points for this distribution. We used two ways to obtain poverty bounds, a parametric and non-parametric approach. The parametric approach assumes that joint distribution is bivariate normal in round 1 and round 2. Non-parametric approach suppose that we make no assumptions about the joint distribution of error term in these regression models. Nonparametric models rely on data-driven estimation procedures that impose relatively few restrictions on the distribution of the underlying data or the functional form of the model in question. However, non-parametric estimation requires several assumptions to hold, chief among them being the smoothness/ continuity of the conditional mean of the dependent variable, the appropriateness of the window width, and the orthogonality of the error term.

Parametric approach provided the relation of household consumption on household’s time invariant characteristics on the base of normality assumption of error terms. Under parametric approach, we assumed *u*_*i*1_ and *u*_*i*2_ follows bivariate normal distribution with correlation coefficient ρ (non-negative) and standard devotions $\sigma_{u_{1}}$ & $\sigma_{u_{2}}$. It provides the estimates of the household who were poor in first round of period but non-poor in second round of period as follows:

$$P^{E}(y_{i1}<z_{1}\;and\;y_{i2}>z_{2})=P(\alpha_{1}^{'}x_{i1}+u_{i1}<z_{1}\;\mathrm{and}\;\alpha_{2}^{'}x_{i2}+u_{i2}>z_{2})$$

$$=\Phi_{2}(\frac{z_{1}-\alpha_{1}^{'}x_{i1}}{\sigma_{u_{1}}},-\frac{z_{2}-\alpha_{2}^{'}x_{i2}}{\sigma_{u_{2}}},-\rho )$$ (18)

Here Φ_2_ (.) indicates the cumulative distribution function (cdf) of bivariate normal distribution and *ϕ*_2_ (.) defines probability density function (pdf).

So, $\rho ,\;\frac{\partial \Phi_{2}(x,y,\rho )}{\partial \rho }=\phi_{2}(x,y,\rho )>0$ (bounded interval: 0,1) and x, y defines the difference between household consumption level and its upper and lower estimates for poverty mobility. The smaller value of ρ indicates the existence of high poverty in the household’s second round of period. Non-parametric poverty bounds estimates corresponding to ρ being equal 1(maximum value) and 0(minimum value).

## 5. Data description

A very concern and mind-numbing process for this study was data extraction. The study used the two rounds of Household Integrated Economic Survey (HIES) conducted by Pakistan Bureau of statistics (PBS) for the years 2010–11 & 2015–16 [25, 26]. The detail about these data sets are given in next.

### 5.1. HIES round 2010–11

This round of HIES survey covered the subsample of 16341 households from 79000 households of survey at district level. A sample of 6589 households is scheduled from urban areas and 9752 households from rural areas. The Survey used the questionnaire of Pakistan Social and Living Standard Measurement (PSLM) with some improvement. The survey gives information about household earnings, health, assets, savings, age, liabilities, education, water facilities, and consumption at state and local level. The survey provides important data on consumption to welfare organizations for analysis of poverty indices.

### 5.2. HIES round 2015–16

This survey round is the seventh round of Household Integrated Economic Survey (HIES) conducted by PSLM project since 2004–05. The data has been collected from 24,238 household including 1605 rural and urban Primary sampling units (PSUs). The Household Integrated Economic Survey (HIES) and Family Budget Survey (FBS) worked together to design the questionnaire. The basic purpose of Family Budget Survey (FBS) was to determine the weight for price statistic. The main purpose of survey is to give information about household earnings, health, assets, savings, age, liabilities, education, water facilities, and consumption at state and local level.

### 5.3. Variables choice

The selection of variables to measure a poverty dimensions, mobility and immobility critically depends on how poverty is currently perceived and apparent. We did the selection of variables with a well normative choice and covers all the basic indicators related to human lives. The selected variables and their detail description is given in Table 2. These variables include household per capita expenditure, household size, gender of household head, age of household head, education level, employment status of head, number of persons studying in home, residence status, facilities at home like toilet, water etc. agricultural property, age ratio of household members and provincial and regional variables. With reference to the present application of this approach is the additional requirement that indicator variables in these models be time invariant. Many time-invariant variables can be readily constructed from the data, such as whether the household head was aged 15 or higher and educated at the primary school level by a particular moment in time. When retrospective data are collected, the range of time-invariant variables can be greatly expanded. Some retrospective variables, such as place of residence at the time of the last survey, are reasonably common in cross-sectional surveys, while other variables, such as sector of work, education level, and occupation at the time of the past survey, could easily be collected retrospectively. Similarly, many time-varying characteristics of the household that can be recalled for round 1 in round 2 [8] are included in the study. For example, whether or not the household head is employed in round 1, and his or her occupation, their place of residence in round 1, etc. Context will also determine the choice of variables to use. In our empirical applications (given in estimation result section) we thus consider a hierarchy of six classes of prediction models which progressively employ more and more data that is sometimes, but not always, collected retrospectively. The summary statistics of the variables are given in S1 Appendix given at the end of the paper.

**Table 2:** Description of variables.

Variables	Definitions
lnPCE	Logarithm of per capita monthly expenditure of household
Hhsize	Total number of member in a household
Mhead	1 for male head
	0 otherwise
Stud_person	1 for member of households is studying
	0 otherwise
H_age	Age of the household head in years
***Head_Emp* *Employment Status of the Household Head***	
	1 for household head self employed
	0 otherwise
***Head_qual* *Qualification of the Household Head***	
	0 Illiterate
	1 Primary to metric
	2 Intermediate
	3 Graduation
	4 Master and above
***Head_resid* *Residential Status of the household head***	
	1 Owner occupied
	0 On rent
Toilet_Facility	1 for Household have toilet facility at home
	0 otherwise
Water_Facility	1 for Household does not pay for drinking water
	0 otherwise
Agri_land	1 for Household having agricultural land
	0 otherwise
***Age ratio of Household members***	
rfage0_4	Age ratio of female household member 0_4 years
rfage5_9	Age ratio of female household member 5_9 years
rfage10_14	Age ratio of female household member 10_14 years
rfage15_55	Age ratio of female household member 15_55 years
rfage56p	Age ratio of female household member 56 plus years
rmage0_4	Age ratio of male household member 0_4 years
rmage5_9	Age ratio of male household member 5_9 years
rmage10_14	Age ratio of male household member 10_14 years
rmage15_55	Age ratio of male household member 15_55 years
rmage56p	Age ratio of male household member 56 plus years (taken as base category)
***Province* *Baluchistan as base category***	
Punjab	1 for Punjab
Sindh	2 for Sindh
KPK	3 for Khyber Pakhtunkhwa
Region	Rural as base category
Urban	1 for urban
Year 2010–11	1 for 2010–11 period (base category)
Year 2015–16	2 for 2015–16 period

Note: Variables are extracting from two surveys round HIES data for Pakistan.

## 6. Estimation results

In this section, we describe the upper and lower bounds of poverty estimates based on synthetic panel data and their OLS regression coefficients. We are interested to provide the poverty bounds for Pakistan in absence of actual panel data. Table 3 presents the estimates when all assumptions are fulfilled, so we will focus here on the interpretation of parametric estimates and then compare it with the nonparametric estimates. We obtained our bounds estimates in the form of three model specifications. Specification 1 where we assume ρ = 1 and ρ = 0 to obtain lower and upper poverty bound. Specification 2 and 3 represent the assumed value of ρ are (0.8, 0.2) and (0.7, 0.3) respectively. The assumed value of ρ in Specification 1 provides most conservative bounds as compared to specification 2 and 3 which provides less conservative bounds. Our results are very encouraging because our estimated bounds sequentially tighter for three specifications as we expected and this naturally comes from trade off the bounds. Our results are consistent with the study of [7, 23], in which they reported that all the estimated bounds sandwich the true rates based on actual panel data with the simulated normally distributed error terms.

**Table 3:** Poverty dynamics estimates from synthetic panel data with simulated error for Pakistan.

Country	Poverty status	Non-parametric bound	Parametric lower bound			Parametric upper bound			Non-parametric bound
			Spec.1 spec.2 spec.3			Spec.3 spec.2 spec.1			
Pakistan	Poor, poor	17.5	20.3	18.3	17.3	13.9	13.2	11.8	12.3
2010–2016									
	Poor, nonpoor	2.5	1.7	3.8	4.7	8.11	8.9	10.3	9.1
	Nonpoor, poor	19	13.7	15.8	16.7	20.1	20.9	22.3	24.3
	Nonpoor, nonpoor	61	64.2	62.2	61.2	57.8	57.1	55.7	54.3
	N	17792	17792	17792	17792	17792	17792	17792	17792

Note: Estimated poverty rates percentage using HIES cross sectional data, second survey round used to obtained predictions. Non-parametric estimates replications are 500. Household head age restricted between 25 and 55 for both survey round.

We are also interested to investigate the transition of poverty for population sub group to check how distant they are from national average. To apply social net policies against poverty, the policy makers don’t have accurate and detail poverty estimates profile [18]. Panel data does not cover all the sub group of population due to limited recourse. The estimates bounds (specification 1, 2 & 3) where all assumptions are met in Table 3 provides the profile of chronic and transitory poor in population sub group for Pakistan. Our estimates show 17% population still remains in poverty as they were in 2010–11. They don’t move in or out of poverty. In the same periods 19% population affected by poverty. The 2.5% poor’s of 2010–11 moves out of poverty in 2015–16 and the percentage of non-poor’s escape from poverty condition in the both rounds of periods are 61 percent.

Table 4 represent the estimates of upper and lower bounds when our data do not meet the assumption of normality. So to overcome the normality issue, we used partial simulated data, we get similar results as we estimated (Table 3) when data fulfilled the normality assumption because we replicate results using data for first survey period. The study [8] provide both results first based on predicted simulated error term of household consumption from bivariate normal distribution. Second they implement this procedure to the actual panel data. For synthetic panel it provides same results. The estimated regression coefficient and standard errors are also same for Tables 3 and 4. The estimates show household characteristics effects on household consumption in the Tables 5–8. Our strategy is based on a linear projection of round 1 consumption onto factors at the person, household, and community levels that are also available in round 2 data. The specification of what is really a forecasting model lacks an evident theory, although there are some diagnostics that can be used as a guide. In general, one should consider factors other than explanatory power (higher R^2^ tends to reduce the variance of prediction error), take into account the statistical significance of parameter estimates (to reduce model error and the ensuing overstatement of mobility), and pay attention to overfitting concerns.

**Table 4:** Poverty dynamics estimates from synthetic panel data for Pakistan with partially simulated errors.

Country	Poverty status	Non-parametric bound	Parametric lower bound			Parametric upper bound			Non-parametric bound
			Spec.1 spec.2 spec.3			Spec.3 spec.2 spec.1			
Pakistan	Poor, poor	17.5	20.3	18.3	17.3	13.9	13.2	11.8	12.3
2010–2016									
	Poor, nonpoor	2.5	1.7	3.8	4.7	8.11	8.9	10.3	9.1
	Nonpoor, poor	19	13.7	15.8	16.7	20.1	20.9	22.3	24.3
	Nonpoor, nonpoor	61	64.2	62.2	61.2	57.8	57.1	55.7	54.3
	N	17792	17792	17792	17792	17792	17792	17792	17792

Note: Estimated poverty rates percentage using HIES cross sectional data, second survey round used to obtained predictions. Non-parametric estimates replications are 500. Household head age restricted between 25 and 55 for both survey round.

**Table 5:** OLS Regression estimates with simulated error 2010–11.

lnPCE	coefficient	Standard error
Hhsize	-0.0704***	0.0082
Mhead	-0.0410*	0.0229
Stud_person	0.0468***	0.0071
H_age	0.0053***	0.0009
Head_emp	-0.008	0.0030
Head_qual	-0.0022	0.0015
Head_resid	0.0803***	0.0217
Toilet_facility	0.1481***	0.0167
Water_facility	0.1463	0.0892
Agri_land	0.2320***	0.0247
rfage0-4	-0.0275***	0.0089
rfage5-9	-0.0801***	0.0068
rfage10-14	-0.0686***	0.0065
rfage15-55	0.0369***	0.0070
rfage56p	0.0505***	0.0158
rrage0-4	-0.0327***	0.0086
rrmage5-9	-0.0854***	0.0063
rmage56p	0.0548***	0.0197
Urban	0.2159***	0.0319
Punjab	-0.0469	0.0494
Sindh	-0.0306	0.0624
Kpk	-0.0525	0.0550
Constant	7.9652***	0.0558

**Note:** Statistical significant is

*** at 0.01

** significant at 0.05

* significant at 0.10

**Table 6:** OLS Regression estimates with simulated error for 2015–16.

lnPCE	coefficient	Standard error
Hhsize	-0.0798***	0.0065
Mhead	-0.0235	0.0198
Stud_person	0.0720***	0.0041
H_age	0.0062***	0.0008
Head_emp	-0.0089**	0.0043
Head_qual	-0.0025*	0.0013
Head_resid	-0.0019	0.0312
Toilet_facility	0.1736***	0.0288
Water_facility	0.2072***	0.0230
Agri_land	0.2507***	0.0206
rfage0-4	-0.0252***	0.0069
rfage5-9	0.0001	0.0067
rfage10-14	-0.0847***	0.0058
rfage15-55	0.0450***	0.0058
rfage56p	0.0664***	0.0151
rrage0-4	-0.0263***	0.0070
rrmage5-9	-0.0931***	0.0067
rmage56p	-0.0931***	0.0197
Urban	-0.1834***	0.0282
Punjab	0.1761**	0.0717
Sindh	0.0924	0.0602
Kpk	0.0908	0.0705
Constant	8.4362***	0.0690

**Note:** Statistical significant is

*** at 0.01

** significant at 0.05

* significant at 0.1

**Table 7:** OLS Regression estimates with partially simulated error 2010–11.

lnPCE	coefficient	Standard error
Hhsize	-0.0704***	0.0082
Mhead	-0.0410*	0.0229
Stud_person	0.0468***	0.0071
H_age	0.0053***	0.0009
Head_emp	-0.008	0.0030
Head_qual	-0.0022	0.0015
Head_resid	0.0803***	0.0217
Toilet_facility	0.1481***	0.0167
Water_facility	0.1463	0.0892
Agri_land	0.2320***	0.0247
rfage0-4	-0.0275***	0.0089
rfage5-9	-0.0801***	0.0068
rfage10-14	-0.0686***	0.0065
rfage15-55	0.0369***	0.0070
rfage56p	0.0505***	0.0158
rrage0-4	-0.0327***	0.0086
rrmage5-9	-0.0854***	0.0063
rmage56p	0.0548***	0.0197
Urban	0.2159***	0.0319
Punjab	-0.0469	0.0494
Sindh	-0.0306	0.0624
Kpk	-0.0525	0.0550
Constant	7.9652***	0.0558

**Note:** Statistical significant is

*** at 0.01

** significant at 0.05

* significant at 0.1

**Table 8:** OLS Regression estimates with partially simulated error 2015–16.

lnPCE	coefficient	Standard error
Hhsize	-0.0798***	0.0065
Mhead	-0.0235	0.0198
Stud_person	0.0720***	0.0041
H_age	0.0062***	0.0008
Head_emp	-0.0089**	0.0043
Head_qual	-0.0025*	0.0013
Head_resid	-0.0019	0.0312
Toilet_facility	0.1736***	0.0288
Water_facility	0.2072***	0.0230
Agri_land	0.2507***	0.0206
rfage0-4	-0.0252***	0.0069
rfage5-9	0.0001	0.0067
rfage10-14	-0.0847***	0.0058
rfage15-55	0.0450***	0.0058
rfage56p	0.0664***	0.0151
rrage0-4	-0.0263***	0.0070
rrmage5-9	-0.0931***	0.0067
rmage56p	-0.0931***	0.0197
Urban	-0.1834***	0.0282
Punjab	0.1761**	0.0717
Sindh	0.0924	0.0602
Kpk	0.0908	0.0705
Constant	8.4362***	0.0690

**Note:** Statistical significant is

*** at 0.01

** significant at 0.05

* significant at 0.10

The four key diagnostics that were performed to assess the regression models are linearity, homoscedasticity, statistical independence, and residual normality. After running the OLS, we individually examined each of these diagnoses using various visualizations. We assessed linearity using the residual versus predicted values, which showed a somewhat consistent trend. To address this problem, we used log transformation dependent variable’s. The homoscedasticity and normality issues were also resolved with the use of this logarithmic transformation. Thus, this model places reasonable demands on the data and is probably applicable to the majority of household surveys. We demonstrated that the error terms may be made more believable by introducing a distributional assumption and more details on the likely plausible range of autocorrelation in these error terms. Log-normality is a reasonable and often used approximation for the distribution of income or consumption, so this condition may hold approximately in practice.

The Table 5 shows the regression estimates of household’s characteristics from the first round of survey (2010–11). The estimates indicate the highly significant effects of household head age, assets (agricultural land), residual status and toilet facility on per capita expenditure (PCE). Household (HH) size and different group male age ratio has also high effects on the PCE. Female HH age ratio 0–14 has less effect on PCE. The HH head education level, employment status has no effect on PCE during 2010–11 period. The gender of HH head has also less effect on PCE. The results also show that HH male age ratio 5–14 has minimum effects on PCE. According to estimates Pakistan urban areas also significantly affect the PCE in 2010–11 years.

The following (Table 6) represents the regression estimates of second round survey (2015–16), that we used in our study. According to results HH size effect on PCE has decreased, and gender of HH head has no effect on PCE during this period. These estimates point out the many aspects related to individual vulnerability to poverty during both survey rounds period. Residence status of HH head effects has also decreased as compare to (Table 5) 2010–11 round estimates. The results also show HH head Agri-land, educational expenditure of HH, toilet facility and water facility has highly effects on PCE. In the estimates of 2010–11 periods water facility did not affect the PCE. It’s also considerable points both periods regression estimates, the female HH age ratio 0–9 and 15-56p has significant effects and male HH age ration 0-56p has decreased. The PCE depends minimally urban areas of Pakistan during 2015–16. The Tables 7 and 8 regression estimates are similar to 5 and 6.

Tables 9 and 10 shows the estimates of regressions on the bases of six classes of models specification for both survey round period. The purpose of these prediction models are used to add more retrospectively data [8]. We start with time invariant independent variables, age of HH head, gender and total person of HH studying. In the second models we introduced urban/rural dummies to capture a HH living at the first time of survey. In the third model included community variables, which can be used from community modules. We obtain this information (poor person group) from HIES survey for both periods and extract the variable on the bases on national poverty cut points. The model 4 represents the added HH work sector, then we further added demographic variable HH size. Finally, we included in the model 6 HH living facilities like water and toilet facility.

**Table 9:** Household consumption estimated parameter, Pakistan 2010–11.

Model 1 Model 2 Model 3 Model 4 Model 5 Model 6						
H_Age11	0.0092***	0.0090***	0.0080***	0.0042	0.0042***	0.0042***
	(0.000)	(0.000)	(0.000)	(0.232)	(0.000)	(0.000)
Mhead11	0.1604***	0.1565***	0.1855***	0.0427***	0.0427***	0.0413***
	(0.000)	(0.000)	(0.000)	(0.000)	(0.000)	(0.000)
Stud_person11	0.1130***	0.1061***	0.1117***	0.0317***	0.0317***	0.0298***
	(0.000)	(0.000)	(0.000)	(0.000)	(0.000)	(0.000)
urban11		0.2387***	0.1866***	0.2223***	0.2223***	0.197***
		(0.000)	(0.000)	(0.000)	(0.000)	(0.000)
gpoorp11			-0.4510***	-0.6113***	-0.6114***	-0.5975***
			(0.000)	(0.000)	(0.000)	(0.000)
Head_emp11				0.0088**	0.0088**	0.0083
				(0.005)	(0.008)	(0.586)
Hhsize11					0.0889***	0.08824***
					(0.000)	(0.000)
Toilet_Facility11						0.1186***
						(0.000)
Water_Facility11 0.1340***						
(0.000)						
Constant 8.9602*** 8.8931*** 8.9950*** 8.9949*** 8.8548*** 8.7664***						
Adjusted R^2^ 0.1886 0.2337 0.3256 0.3256 0.4785 0.4851						
σ 0.4934 0.4795 0.4498 0.4498 0.3956 0.3931						
N 16341 16341 16341 16341 16341 16341						

Note

*p< 0.1

**p< 0.05, and

***p< 0.01.

**Table 10:** Household consumption estimated parameter, Pakistan 2015–16.

Model 1 Model 2 Model 3 Model 4 Model 5 Model 6						
H_age16	0.0001***	0.1102***	0.0097***	0.0097	0.0054***	0.0054***
	(0.000)	(0.000)	(0.000)	(0.000)	(0.000)	(0.000)
Mhead16	0.1235***	0.1008***	0.1458***	0.1448***	0.0314***	0.0315***
	(0.000)	(0.000)	(0.000)	(0.000)	(0.000)	(0.000)
Stud_person16	0.1237***	0.1179***	0.1243***	0.1458***	-0.4324***	0.4329***
	(0.000)	(0.000)	(0.000)	(0.000)	(0.000)	(0.000)
urban16		-0.273***	-0.1739***	0.1243***	-0.1996***	- 0.2000***
		(0.000)	(0.000)	(0.000)	(0.000)	(0.000)
gpoorp16			-0.5023***	-0.1739***	-0.6549***	-
			(0.000)	(0.000)	(0.000)	0.6548***
						(0.000)
Head_emp16				0.5023	0.0027	0.0027
				(0.717)	(0.392)	(0.329)
Hhsize16					0.0828***	0.0829***
					(0.000)	(0.000)
Toilet_Facility16						0.0057
						(0.548)
Water_Facility16 0.0185						
(0.382)						
Constant 9.4669*** 9.5950*** 9.6738*** 9.6737*** 9.6224*** 9.6171***						
Adjusted R^2^ 0.2058 0.2549 0.3676 0.3675 0.4905 0.4905						
σ 0.5810 0.5017 0.4622 0.46218 0.4148 0.4148						
N 16341 16341 16341 16341 16341 16341						

Note

*p< 0.1

**p< 0.05, and

***p< 0.01.

The comparison of both the estimated survey models show that the HH head employment status, HH toilet and water facility affects is high in first round and has no effect these are captured in second round. In both periods, group of poor person has minimum effect on PCE. Models also captured the high effect of HH size, head age, no of person studying and head gender on PCE in both rounds of surveys. This analysis of these estimates indicates that head age, HH size and head gender can be more concern for HH PCE.

## 7. Conclusion and recommendations

In this study we used repeated cross-sections methodology to construct the synthetic panel data for Pakistan from existing household consumption survey. Our study provided the poverty bounds, chronic and transitory poor’s measures during 2010–16 periods. We also test the performance of different interval length between the two rounds of cross sectionals and explored the prospects of some insights into mobility across poverty lines and poverty duration.

These aspects can make this method more successful: under non-parametric approach how well one capture the relation between dependent and independent variable. Under parametric approach how we capture accurate autocorrelation range for error term. From the results, it is noticed that estimates accuracy increases when poverty bounds turn into narrow, when we have opportunity to apply previous period information on household demographic characteristics. The parametric estimates can also improve, when we can predict better dependent variable.

Most of the developing countries are facing the unavailability of national represented longitude data. When data are seldom available, the collected sample are often small with limited duration. The collection of national representative panel data is very costly and time taking; it can pose capacity-related challenges for under developing countries. To construct an even simple description about household movement into or out of poverty, individual’s consumption dynamic information is required.

Poverty is a global issue of concern, affected most of developing countries not only physically but also psychologically. Poverty divide a nation. The policy makers do care about accurate information on poverty transition so they can design reliable policies. Future research could thus focus on extending the list of empirically estimated correlation terms by looking at panel data from under develop countries, as well as creating a similar list for other welfare outcomes and can compare the results of these techniques with panel data. These typologies of the range of autocorrelation for the error terms could then be used to provide estimates for countries with similar settings. Another promising direction is to collect data on a smaller subpanel (i.e., for cost savings) and combine the estimated correlation terms from this subpanel with larger cross section samples to estimate poverty transitions based on our parametric approach.

## 8. Limitations

There are many limitations about the implication of these methods. Firstly, genuine panel data is very rare in developing world and particularly in Pakistan. This has limited the feasibility of constructing even relatively simple description of transitions in and out of poverty, while policy makers do care about these transitions. Secondly, information about some variables such as demographic composition of households is collected concurrently but not retrospectively. So, there may be a scope of collecting such data going forward. When certain shocks such as development of chronic illness or death of a spouse occur, since such variables might also help predict poverty status. Thirdly, the whole analysis has been based on one particular poverty line for each year.

## Supporting information

S1 Dataset (XLSX)

S1 Appendix (DOCX)
