# Supplementary material for: Estimation of poverty bounds for Pakistan using synthetic panel data
Source: PLoS One. 2023 Mar 23;18(3):e0276673. doi: 10.1371/journal.pone.0276673 (PMC10035873; doi:10.1371/journal.pone.0276673)
Supplement: S1 Appendix — (DOCX) [file pone.0276673.s002.docx]

**Appendix:**

**Table 1 :Summary Statistics of the Study variables**

|  | **2010** | | | **2015** | | |
| --- | --- | --- | --- | --- | --- | --- |
|  | **Mean** | | **Standard Deviation** | **Mean** | | **Standard Deviation** |
| PCME | 3380.579 | | 2988.284 | 3546.985 | | 2756.92 |
| **Proportion of household members of different age brackets to total household size and Excluded Category is female of 56 years of age and over** | | | | | | |
| HHsize | 6.410 | 2.591 | | 6.814 | 2.862 | |
| ***Household head characteristics*** |  |  | |  |  | |
| Mhead | 0.084 | 0.278 | | 0.075 | 0.263 | |
| Stud_person | 0.456 | 0.342 | | 0.568 | 0.389 | |
| H_age | 45.999 | 13.122 | | 45.573 | 13.746 | |
| E_Status | 0.190 | 0.392 | | 0.266 | 0.442 | |
| Head_Illiterate | 0.297 | 0.457 | | 0.011 | 0.104 | |
| Head_Primary to metric | 0.635 | 0.481 | | 0.399 | 0.490 | |
| Head_Intermediate | 0.645 | 0.479 | | 0.517 | 0.500 | |
| Head_Graduate | 0.171 | 0.377 | | 0.137 | 0.346 | |
| Head_resid | 0.239 | 0.344 | | 0.328 | 0.356 | |
| Agriculture_Landowner | 0.080 | 0.271 | | 0.092 | 0.289 | |
| Water_facility | 0.845 | 0.362 | | 0.859 | 0.348 | |
| Toilet_facility | 0.139 | 0.346 | | 0.256 | 0.436 | |
| rm0_4 | 0.055 | 0.099 | | 0.062 | 0.102 | |
| rm5_9 | 0.070 | 0.106 | | 0.071 | 0.104 | |
| rm10_14 | 0.064 | 0.100 | | 0.063 | 0.097 | |
| rm15_55 | 0.276 | 0.168 | | 0.258 | 0.163 | |
| rm56P | 0.045 | 0.095 | | 0.048 | 0.099 | |
| rf0_4 | 0.057 | 0.100 | | 0.060 | 0.101 | |
| rf5_9 | 0.062 | 0.099 | | 0.065 | 0.099 | |
| rf10_14 | 0.055 | 0.093 | | 0.058 | 0.094 | |
| rf15_55 | 0.277 | 0.151 | | 0.271 | 0.147 | |
| rf56p | 0.040 | 0.100 | | 0.042 | 0.103 | |
| ***Region*** |  |  | |  |  | |
| Urban | 0.423 | 0.494 | | 0.403 | 0.491 | |
| Rural | 0.577 | 0.494 | | 0.597 | 0.491 | |
| ***Provinces*** | | | | | | |
| Punjab | 0.431 | 0.495 | | 0.439 | 0.496 | |
| Sindh | 0.234 | 0.423 | | 0.246 | 0.430 | |
| KPK | 0.186 | 0.389 | | 0.185 | 0.388 | |
| Baluchistan | 0.149 | 0.357 | | 0.131 | 0.337 | |
| **N** | 16290 | | | 24238 | | |
